# Restoration of Ankle Stability After Complete Traumatic Loss of the Distal Fibula Using Peroneus Brevis Tendon Reconstruction Without Osseous Reconstruction

**DOI:** 10.7759/cureus.102499

**Published:** 2026-01-28

**Authors:** Siddhart Yadav, Purvesh Bhrambhatt, Akash Jadon, Aashish Pathak, Dharm Bedwal

**Affiliations:** 1 Orthopaedics and Trauma, Apollo Hospitals, Navi Mumbai, IND

**Keywords:** distal fibula loss, lateral ligament reconstruction, peroneus brevis tendon, post-traumatic ankle injury, without osseous reconstruction

## Abstract

The distal fibula plays a key role in ankle stability by forming the ankle mortise and serving as the attachment site for the lateral ligament complex. Traumatic loss of the distal fibula is extremely rare and is associated with ankle instability, valgus deformity, and eventual arthritis if not addressed.

We report the case of a 34-year-old female who sustained a high-energy open injury with complete loss of the distal fibula, disruption of the lateral ligament complex, and a comminuted distal tibial fracture following a road traffic accident. Initial management included emergency debridement, application of a joint-spanning external fixator, and serial vacuum-assisted closure (VAC) dressings. Definitive treatment involved intramedullary tibial nailing with tricortical iliac crest bone grafting, reconstruction of the lateral ligament complex using a peroneus brevis tendon split without osseous reconstruction of the distal fibula, and soft-tissue coverage with a gracilis free flap.

At two-year follow-up, the patient was pain-free, demonstrated no clinical or radiographic signs of ankle instability, and had returned to full activity without functional limitations.

Lateral ligament reconstruction using the peroneus brevis tendon can provide satisfactory ankle stability and function even in the absence of distal fibular reconstruction, when combined with appropriate bony stabilization and soft-tissue coverage. Long-term follow-up is necessary to monitor for potential late degenerative changes.

## Introduction

The distal fibula plays a critical role in maintaining ankle stability, not only through its articulation with the talus at the ankle mortise [1-4] but also as the site of attachment for the lateral ligament complex. This ligamentous complex is essential for stabilizing the ankle joint during external rotation and inversion stresses [5]. Traumatic loss of the distal fibula leads to significant ankle instability, progressive valgus deformity, and, if left untreated, eventual degenerative arthritis [6].

We present a case of a high-energy open injury with post-traumatic loss of the distal fibula and lateral ligament complex, associated with a comminuted distal tibial fracture, in a 34-year-old female. To the best of our knowledge, only two similar cases of post-traumatic distal fibular loss have been reported in the literature [7]. In contrast, distal fibular resection is more commonly described in oncologic settings as part of limb-salvage procedures for malignant tumors.

Given the rarity of post-traumatic cases, there is no standardized treatment protocol, and various reconstructive techniques have been reported. These include reconstruction of the distal fibula with reversed proximal fibular grafts to restore ankle mortise anatomy, autologous iliac crest bone grafting (with or without lateral ligament reconstruction) to address segmental bone loss but with potential donor-site morbidity, and ankle arthrodesis as a salvage option that reliably restores stability at the cost of ankle motion [8-10]. Each approach carries its own benefits and limitations, and surgical decision-making must be individualized, considering the extent of bone loss, ligamentous injury, and soft-tissue status.

This case report aims to describe the surgical technique and short-to-mid-term functional outcome of lateral ligament reconstruction using the peroneus brevis tendon in the absence of distal fibular reconstruction.

## Case presentation

A 34-year-old female sustained a high-energy road traffic injury while traveling in a three-wheeled vehicle. She was seated on the passenger side when the vehicle overturned, resulting in entrapment of her left ankle between the vehicle and the road surface. She was promptly transported to our hospital for evaluation and management.

**Figure 1:**
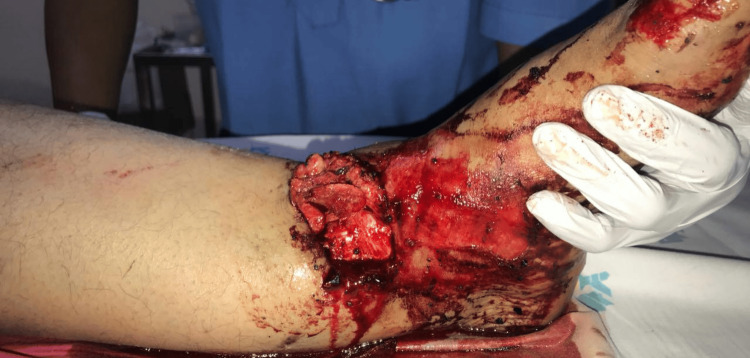
Clinical picture taken in the emergency room

On presentation, she had a severe soft-tissue injury involving the left ankle and leg. Clinical examination revealed a complete loss of skin and crushed muscle over the lateral aspect, measuring approximately 10×5×5 cm, with gross contamination by soil. Additionally, there was a contused lacerated wound over the medial aspect of the leg, measuring 10×2×2 cm and extending to the bone (Figure 1). Peripheral vascular assessment revealed a palpable dorsalis pedis pulse and a feeble posterior tibial artery pulsation.

**Figure 2:**
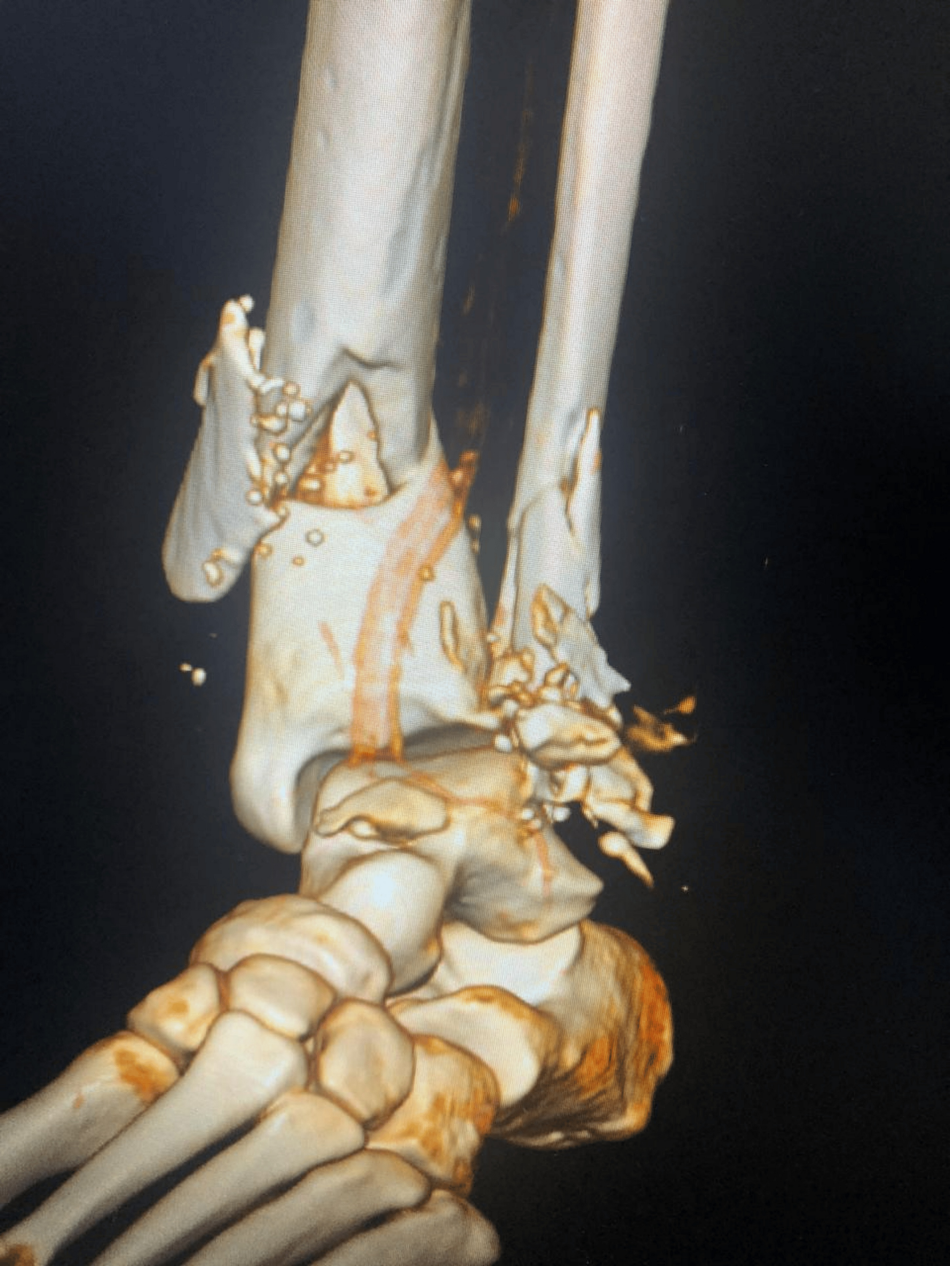
Three-dimensional CT scan view showing comminuted distal fibula fracture and distal shaft tibia fracture

CT angiography demonstrated adequate flow in the anterior tibial artery with reduced flow in the posterior tibial artery. Radiographs and CT scans confirmed skeletal injuries, which included loss of the lateral malleolus, a comminuted distal fibula fracture, and a comminuted distal tibial shaft fracture (Figure 2).

Immediate surgical management

**Figure 3:**
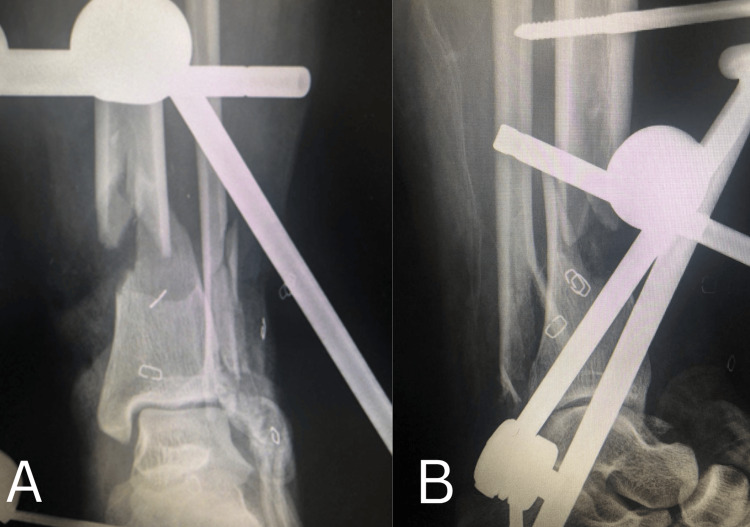
Immediate postoperative radiographs after applying an ankle-spanning external fixator A. Anteroposterior view demonstrating application of a spanning external fixator with restoration of length and alignment of the distal tibia. B. Lateral view confirming satisfactory provisional reduction and maintenance of sagittal alignment of the ankle joint.

The patient underwent emergency debridement under anesthesia. Intraoperatively, the posterior tibial artery was found to be intact. All loose bone fragments were removed, and thorough debridement of devitalized tissue was performed. A joint-spanning external fixator was applied (Figure 3). Primary closure of the medial contused lacerated wound was achieved, while the lateral aspect revealed complete loss of the lateral malleolus and disruption of the lateral ligament complex of the ankle. The anterior talofibular ligament (ATFL) was torn, whereas the peroneal tendons were intact. Given the extensive soft-tissue loss laterally, a vacuum-assisted closure (VAC) dressing was applied.

Following three sequential VAC dressing changes, the lateral wound appeared healthy and ready for definitive management.

Definitive surgical management

The patient subsequently underwent interlocking tibial nailing with tricortical bone grafting and reconstruction of the lateral ligament complex using the peroneus brevis tendon, without osseous reconstruction of the lateral malleolus. Soft-tissue coverage was achieved with a gracilis free flap.

Surgery was performed under combined spinal and epidural anesthesia. The tibia was first stabilized with an intramedullary tibial nail, with both proximal and distal locking bolts placed. The bone defect of approximately 1 cm was filled with a tricortical iliac crest bone graft harvested from the ipsilateral side. Ankle stability was assessed under an image intensifier and found to be grossly unstable with marked varus opening.

Reconstruction of the lateral ligament complex of the ankle was performed without osseous reconstruction of the lateral malleolus, using the peroneus brevis tendon. The tendon was split at the myocutaneous junction, and the proximal portion was reflected distally. With the ankle held in 30° of plantarflexion, the calcaneofibular ligament portion was reconstructed and sutured with Ethibond no. 5 under appropriate tension. The ankle was then positioned in neutral, and the remaining portion of the tendon was reflected anteriorly and sutured to reconstruct the talocalcaneal portion of the ligament.

The peroneus brevis tendon was then reflected proximally and fixed to the tibia approximately 2 cm proximal to the joint line using a 3.5 mm cortical screw and Ethibond no. 5. The anterior ankle capsule and ATFL were repaired using the peroneus brevis tendon with the ankle maintained in 30° of dorsiflexion.

**Figure 4:**
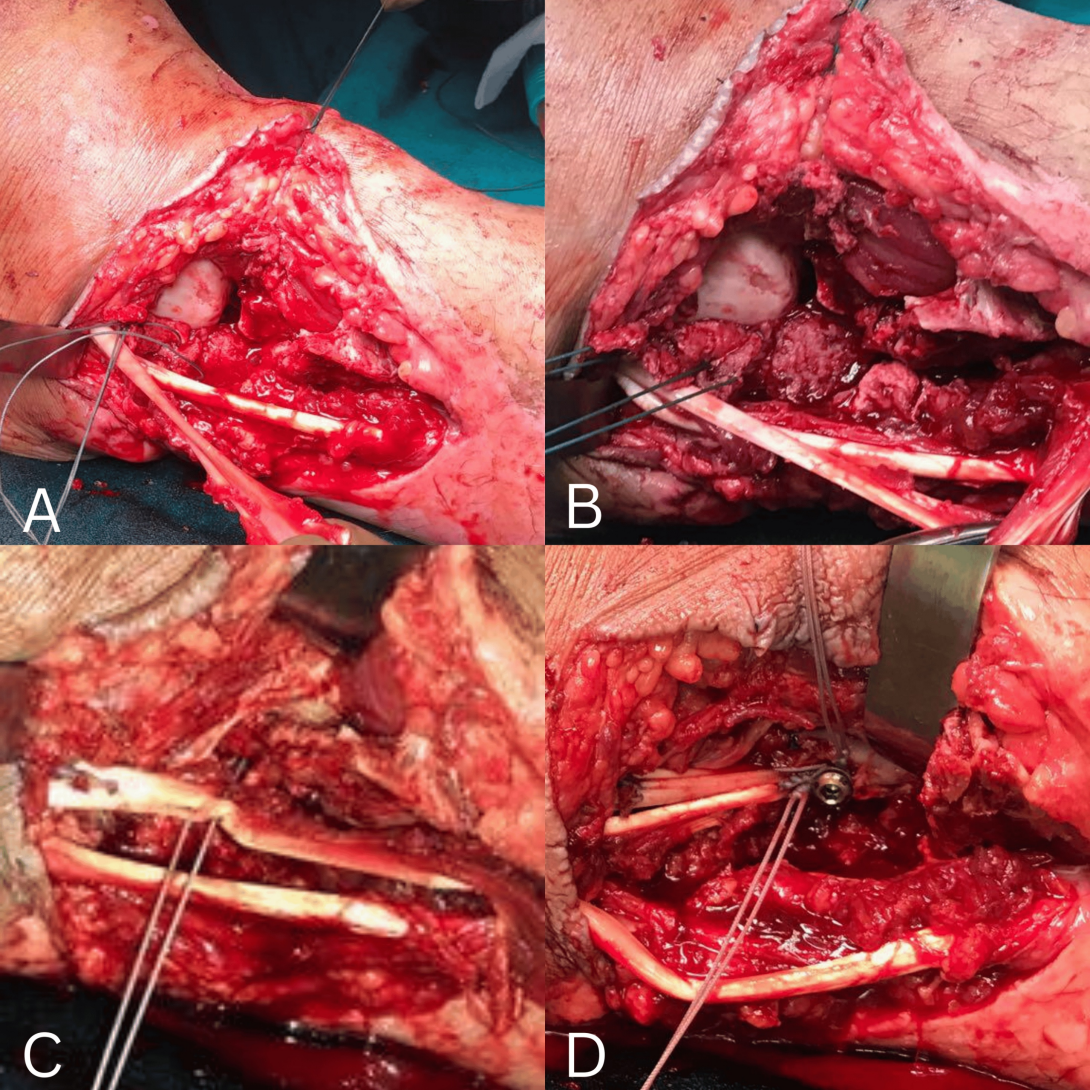
Intraoperative images showing lateral ligament reconstruction using the peroneus brevis tendon A. Isolation of peroneus brevis tendon. B. Proximal portion of tendon reflected distally. C. Reconstruction of calcaneofibular ligament. D. Peroneus brevis tendon reflected proximally and fixed to the tibia with a 3.5 mm screw and Ethibond.

**Figure 5:**
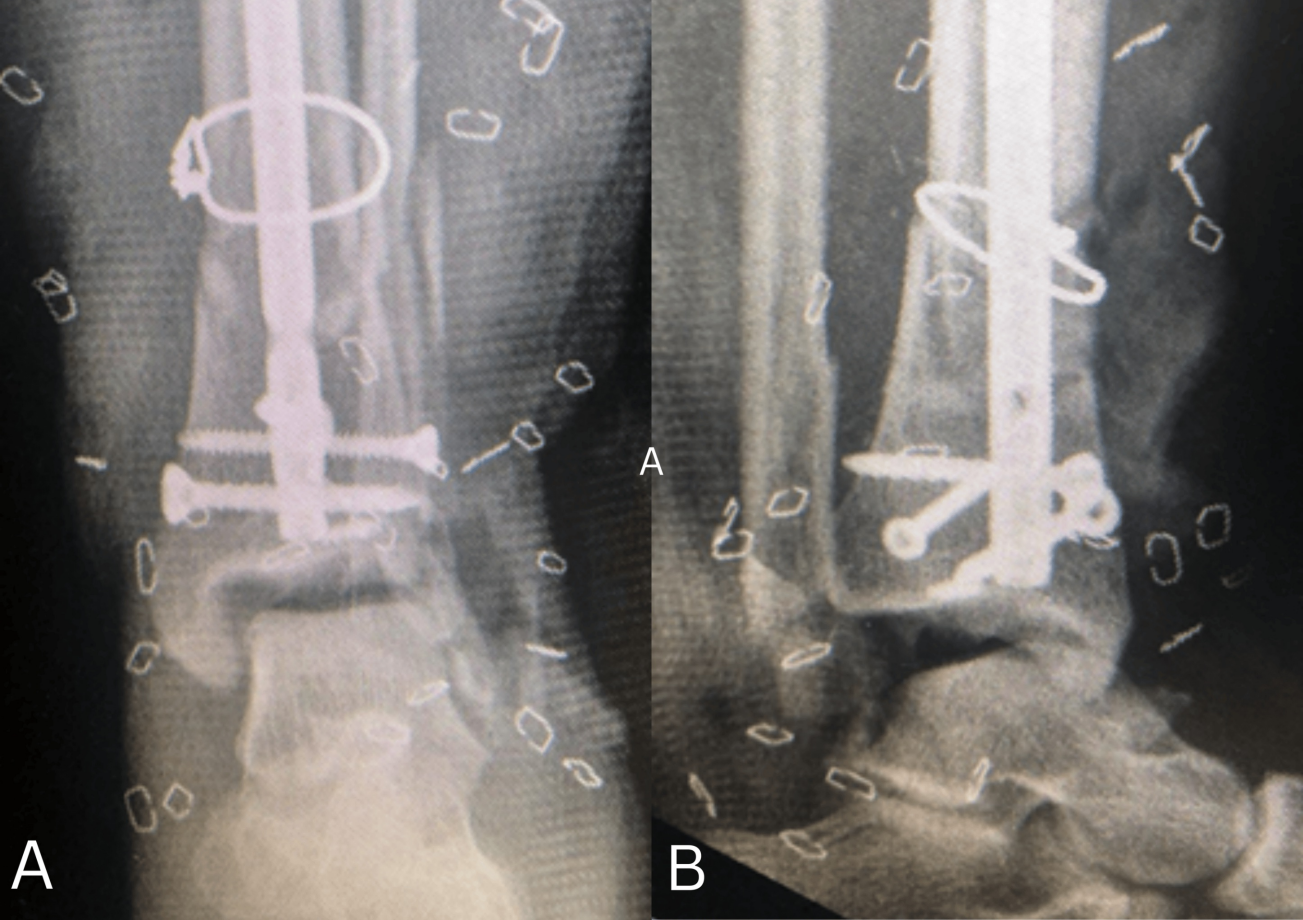
Immediate postoperative radiographs A. Anteroposterior view demonstrating an intramedullary tibial nail with distal interlocking screws and cerclage wiring, with maintained alignment. B. Lateral view confirming appropriate hardware positioning and satisfactory sagittal alignment of the distal tibia and ankle.

Soft-tissue reconstruction of the lateral aspect was achieved using a vascularized gracilis free flap, which was subsequently covered with a split-thickness skin graft (Figure 4 and Figure 5).

Postoperative care and outcome

**Figure 6:**
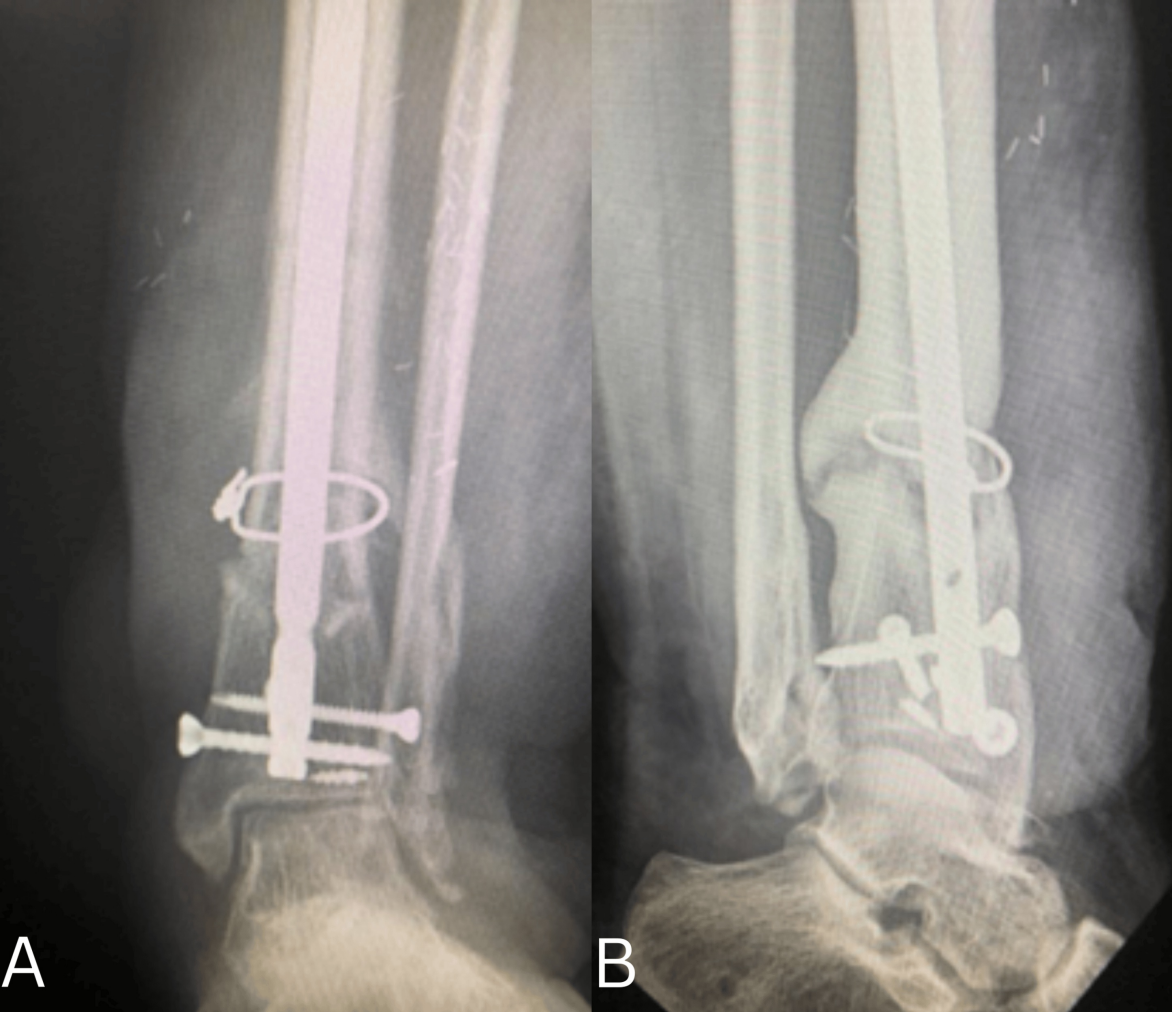
Postoperative follow-up at six months (weight-bearing views) A. Anteroposterior view demonstrating progressive fracture healing with bridging callus formation and maintained alignment. B. Lateral view confirming continued consolidation of the fracture with satisfactory sagittal alignment and no evidence of secondary displacement.

The ankle was immobilized in a posterior plaster slab for six weeks, and the patient was kept non-weight-bearing during this period. At three and six months postoperatively, ankle stability was confirmed on weight-bearing radiographs, which demonstrated no talar tilt or anterior talar translation (Figure 6).

**Figure 7:**
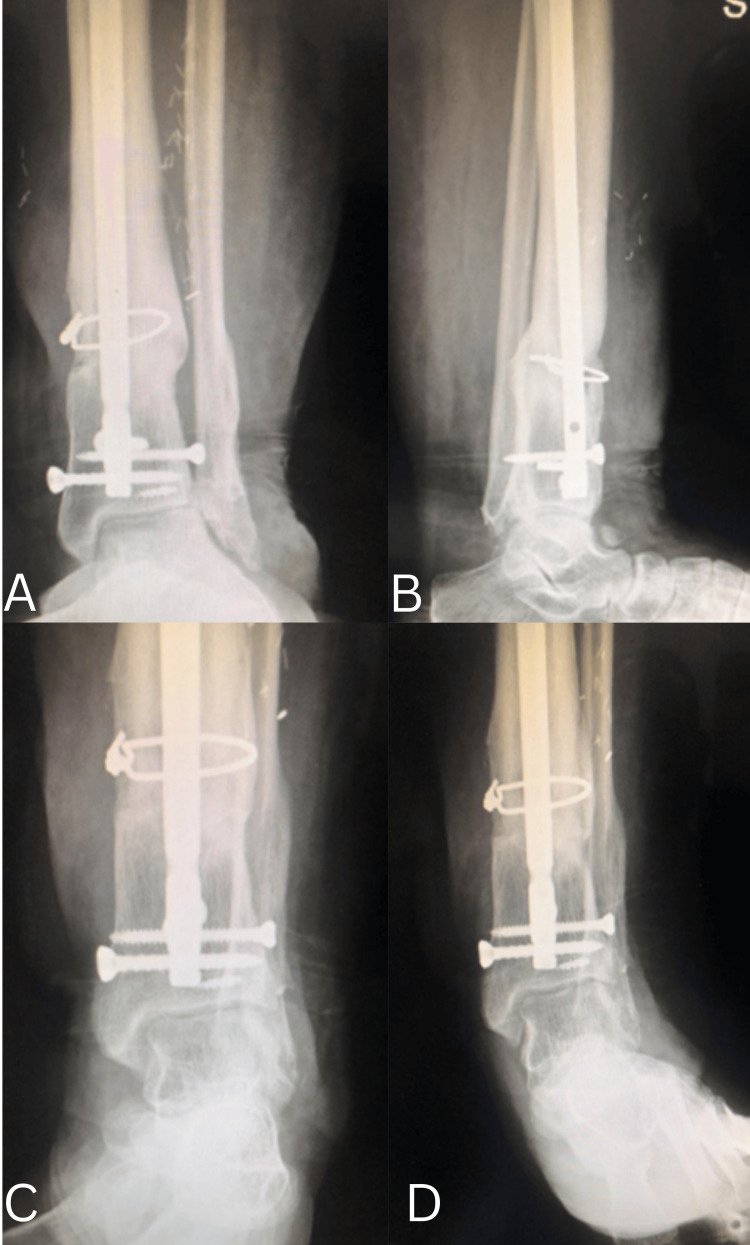
Two-year follow-up radiographs of the distal tibia and ankle A. Anteroposterior view demonstrating complete fracture union with mature callus formation and maintained alignment. B. Lateral view confirming solid consolidation of the fracture with preserved sagittal alignment. C. Varus stress view showing a stable ankle mortise without evidence of instability. D. Valgus stress view demonstrating maintained joint congruency with no medial or lateral talar tilt.

**Figure 8:**
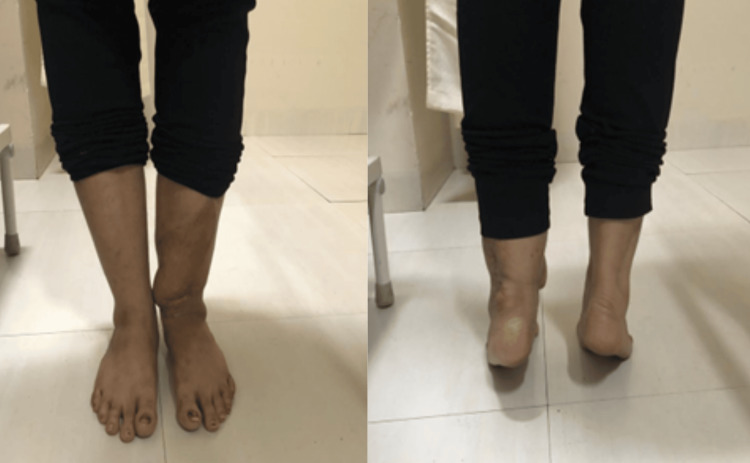
Clinical picture at two-year follow-up

At two-year follow-up, the patient reported no pain or instability and was able to perform all activities, including running and stair climbing, without difficulty. Radiographs confirmed solid union of the tibial fracture and a well-aligned, stable ankle joint (Figure 7 and Figure 8).

## Discussion

The lateral malleolus plays a crucial role in ankle biomechanics, carrying approximately one-sixth of the static load of the leg as it articulates with the talus during longitudinal weight transmission. In addition, the lateral ligament complex provides dynamic stability, preventing excessive varus or inversion stress at the ankle joint.

Complete traumatic loss of the distal fibula is rare and typically occurs as a result of high-velocity injuries. While resection of the distal fibula is a recognized treatment option in oncologic conditions involving fibular tumors, post-traumatic loss of the lateral malleolus remains an exceedingly uncommon clinical scenario. Reconstruction of the distal fibula, when indicated, is technically demanding, and multiple strategies have been described, including proximal fibular grafts (vascularized or non-vascularized), autologous iliac crest bone grafting, neurocutaneous flaps, and even ankle arthrodesis. Each technique carries its own benefits and limitations, including donor-site morbidity, risk of nonunion, and potential compromise of joint mobility.

Failure to reconstruct the lateral ligament complex after distal fibular resection has been associated with valgus deformity, progressive talar tilt, and eventual development of degenerative ankle arthritis. Therefore, restoration of ankle stability through ligament reconstruction is strongly recommended. Shi et al. [5] reported two cases of post-traumatic distal fibular loss managed with neurocutaneous flaps and no bony reconstruction. Both patients had intact tibiae and preserved medial stability, and they achieved satisfactory outcomes, suggesting that ankle stability may be partially maintained by flap stiffness, deep fascial adherence, and intact medial ligamentous structures.

In contrast, our patient had a concomitant comminuted distal tibial fracture, resulting in compromised medial stability. Therefore, we elected to perform lateral ligament complex reconstruction to restore joint stability, despite not performing osseous reconstruction of the distal fibula. Anatomical reconstruction was not feasible due to the absence of the lateral malleolus. Instead, we opted for a non-anatomical reconstruction using the peroneus brevis tendon, a well-described technique for chronic lateral ankle instability that offers the advantages of minimal donor-site morbidity, avoidance of additional bony tunnels, and reduced risk of further osseous trauma.

Soft-tissue coverage was another major challenge in this case due to extensive lateral skin and muscle loss. A gracilis free flap with split-thickness skin grafting was utilized to achieve durable coverage, and this approach provided a stable, well-vascularized bed conducive to healing and infection prevention.

At two-year follow-up, our patient demonstrated a pain-free, stable ankle joint with no evidence of talar tilt, lateral subluxation, or early post-traumatic arthritis. These findings suggest that lateral ligament reconstruction without osseous reconstruction of the distal fibula can provide adequate functional and radiological outcomes in carefully selected cases, even in the setting of concomitant tibial fractures. Nonetheless, longer-term follow-up is required to confirm the durability of these results and monitor for potential late degenerative changes.

## Conclusions

This case highlights that traumatic loss of the distal fibula, although rare, can be successfully managed without osseous reconstruction when lateral ligament stability is restored using the peroneus brevis tendon and supported by adequate bony fixation and soft-tissue coverage. Our patient achieved a stable, pain-free ankle with full functional recovery at two years, demonstrating that this approach may be a viable option in carefully selected cases. Continued long-term follow-up is necessary to monitor for potential late degenerative changes and to further define the role of ligament-based reconstruction in the absence of the distal fibula.

## References

[1] Norman-Taylor FH, Sweetnam DI, Fixsen JA (1994). Distal fibulectomy for Ewing's sarcoma. J Bone Joint Surg Br.

[2] Babhulkar SS, Pande KC, Babhulkar S (1995). Ankle instability after fibular resection. J Bone Joint Surg Br.

[3] Jones RB, Ishikawa SN, Richardson EG, Murphy GA (2001). Effect of distal fibular resection on ankle laxity. Foot Ankle Int.

[4] Uchiyama E, Suzuki D, Kura H, Yamashita T, Murakami G (2006). Distal fibular length needed for ankle stability. Foot Ankle Int.

[5] Carrell WB (1938). Transplantation of the fibula in the same leg transposition of the fibula for loss of the tibial diaphysis. J Bone Joint Surg.

[6] Beris AE, Lykissas MG, Korompilias AV, Vekris MD, Mitsionis GI, Malizos KN, Soucacos PN (2011). Vascularized fibula transfer for lower limb reconstruction. Microsurgery.

[7] Bibbo C, Ehrlich DA, Kovach SJ 3rd (2015). Reconstruction of the pediatric lateral malleolus and physis by free microvascular transfer of the proximal fibular physis. J Foot Ankle Surg.

[8] Gao YS, Zhang CQ, Sheng JG (2016). Reverse transfer of the proximal vascularized fibula to reconstruct the lateral malleolus: a case report and literature review. J Foot Ankle Surg.

[9] Herring CL Jr, Hall RL, Goldner JL (1997). Replacement of the lateral malleolus of the ankle joint with a reversed proximal fibular bone graft. Foot Ankle Int.

[10] de Gauzy JS, Kany J, Cahuzac JP (2002). Distal fibular reconstruction with pedicled vascularized fibular head graft: a reconstruction with pedicled vascularized fibular head graft: a case report. J Pediatr Orthop B.

